# Gut microbiome, metabolome and alopecia areata

**DOI:** 10.3389/fmicb.2023.1281660

**Published:** 2023-11-15

**Authors:** Zhiyu Liu, Xiaoyan Liu

**Affiliations:** ^1^School of Medicine of Zhejiang University, Hangzhou, China; ^2^Department of Dermatology, The First Affiliated Hospital, School of Medicine of Zhejiang University, Hangzhou, China

**Keywords:** alopecia areata, gut microbiome, metabolome, JAK–STAT signaling, TGF-β signaling, Wnt/β-catenin signaling, oxidative stress

## Abstract

Alopecia areata (AA) is a type of dermatological disease characterized by rapid and non-scarring hair loss of the scalp or body skin that may be related to genetic, immunological and physiological factors. It is now believed that AA is associated with oxidative stress, autoimmune disease, neuropsychological factors, pathogens, immune checkpoint inhibitors and microecological imbalance under the premise of host genetic susceptibility. In recent years, studies have revealed the significant role of the gut microbiome or metabolome in many aspects of human health. Diverse studies have revealed that the gut microbiome and metabolome have an important influence on skin conditions. This review highlights the relationship between AA and the gut microbiome or metabolome to provide novel directions for the prevention, clinical diagnosis and treatment of AA.

## 1. Introduction

Alopecia areata (AA) is a type of autoimmune inflammatory dermatological disease characterized by non-scarring hair loss of the scalp or body skin (Zhou et al., 2021). Nearly 2% of the general population undergo AA at some point during their lifetime (Pratt et al., 2017). Although some patchy AA are self-limited, a large proportion of AA are typically relapsing and severe. In these patients, their hair loss is extensive and the course is persistent, which seriously affect their physical and psychological health (Pratt et al., 2017). In pathogenesis, increasing lines of evidence support the notion that under the premise of host genetic susceptibility, AA is triggered by disturbance of inflammatory pathway, oxidative stress, neuropsychological factors and pathogens, companying with comorbidities and microecological imbalance (Alessandrini et al., 2021).

In recent years, studies have revealed the significant role of the gut microbiome or metabolome in many aspects of human health (Gomaa, 2020). Diverse studies have revealed that the gut microbiome and metabolome have an important influence on skin conditions and inflammatory skin diseases (Mahmud et al., 2022), including AA (Sanchez-Pellicer et al., 2022a,b). Especially, fecal microbiota transplantation (FMT) and therapies that alter the gut microbiota or metabolites potentiate the effect of immunotherapies (Baruch et al., 2021; Huang J. et al., 2022), indicating promising application of therapies targeting gut microbiome or metabolome. Brzychcy et al. (2022) revealed a loss of overall richness and a decrease in taxonomic diversity across all the stool samples of the AA patients compared to healthy individuals. While other studies had found no significant changes in α or ß diversity of the gut microbiota structure of AA patients. However, they still hold the opinion that intestinal bacterial biomarkers associated with AA or part of the gut microbial communities should be further studied as they may be involved in the pathophysiology of AA (Moreno-Arrones et al., 2020; Lu et al., 2021; Rangu et al., 2021).

In this review, we systematically evaluate current data regarding gut microbiome, metabolites and their effects on AA. We discuss potential mechanisms of the gut-skin axis in AA and the link between the gut microbiota or metabolites and oxidative stress (OS) or inflammatory pathways, such as Janus kinases and signal transducers and activators of transcription (JAK–STAT) signaling, transforming growth factor beta (TGF-β) signaling, and Wnt/β-catenin signaling. This review will increase our understanding of the impacts of gut microbiome on AA to aid in finding new medications for AA.

## 2. Effects of the gut-skin axis and gut metabolites on skin and hair

The existence of the microbiota-gut-brain axis (Cryan et al., 2019) and the gut-brain-skin axis has been investigated and studied in recent years (Mahmud et al., 2022; Sanchez-Pellicer et al., 2022a,b). These two axes play a crucial role in modulating intestinal health, emotional state and inflammation in the human body and skin (Sanchez-Pellicer et al., 2022a,b).

The gut microbiota and gut metabolites regulate both the innate and adaptive immune systems, which in turn influence the homeostasis of the skin (Sinha et al., 2021; Sanchez-Pellicer et al., 2022a,b). Numerous studies have shown that there is a bidirectionality between the gut microbiota and skin homeostasis. Dybiosis in the intestinal microbiome is linked to the development of skin diseases, such as psoriasis, acne vulgaris, atopic dermatitis, and even skin cancers (De Pessemier et al., 2021). Genes affecting gut microbial colonization may induce the Th1 response, leading to the production of interferon (IFN), which signals through the JAK–STAT pathway and then causes abnormal growth of hair follicle cells and ultimately the development of alopecia (Simakou et al., 2019).

## 3. The pathogenesis of alopecia areata

Although the pathogenesis of AA remains unclear, it is generally believed to be primarily related to loss of hair bulb privilege and autoimmune responses (Strazzulla et al., 2018; Olayinka and Richmond, 2021). AA has been identified as an organ-specific autoimmune disease of the hair follicle with a genetic background (Trueb and Dias, 2018; Olayinka and Richmond, 2021). In recent decades, new insights into the genetics, epigenetics, oxidative stress, autoimmune comorbidities, psychologic stress, and microbiome or metabolome of AA, have updated the etiopathogenetics of AA. These factors work together through several signaling pathways and collectively contribute to AA.

### 3.1. JAK–STAT signaling pathways

In AA, CD8 + NKG2D + T cells are activated by the stimulators mentioned above, and IFN-γ is produced via the JAK1 and JAK3 pathways, which further promote IL-15 production in follicular epithelial cells via JAK1 and JAK2. Then, IL-15 inversely combines with CD8 + NKG2D + T cells amplify a positive feedback loop that results in the IFN-γ storm (Olayinka and Richmond, 2021; Zhou et al., 2021). In addition, the JAK–STAT pathway can suppress the proliferation and activation of hair stem cells and reduce angiogenesis, therefore plays a part in the premature termination of the anagen phase in AA. Based on these discoveries, JAK inhibitors have been used as a new strategy for the clinical treatment of AA (Sterkens et al., 2021). After binding with JAK, the inhibitor makes it unable to bind and activate STAT and thereby inhibits the entry of the latter into the nucleus for transduction of cytokines, such as IFN-γ, IL-2, IL-7, IL-15 and IL-21, which play a crucial role in the pathogenesis of AA (Montilla et al., 2019).

### 3.2. TGF-β signaling pathway

TGF-β is a pleiotropic cytokine with regulatory and inflammatory activities (Waskiel-Burnat et al., 2021). TGF-β1 functions as a negative regulator of cell growth, inhibiting epithelial cell growth and influencing the functioning of the immune system by acting as a keratinocyte proliferation inhibitor and apoptosis inducer. In 2015, a genome-wide meta-analysis in AA uncovered new molecular pathways disrupted in AA, including JAK–STAT signaling, autophagy/apoptosis and TGF-β signaling, the latter functioning to induce the differentiation of Tregs that participate in hair biology (Betz et al., 2015). Many experiments and studies have evaluated the change in the serum level of TGF-β in patients with AA. Some results have shown that the serum level of TGF-β in patients with AA is higher than that in healthy controls (Manimaran et al., 2022) whereas others showed the opposite (Tembhre and Sharma, 2013; Alzolibani et al., 2016). Due to these conflicting results, further studies on the role of TGF-β in AA are needed.

Studies revealed that there is a crosstalk between insulin-like growth factor-1 (IGF-1) and TGF-β. Cogent evidence supports that the negative role of TGF-β in cell growth could be suppressed by the complicated influence of the IGF-1 signaling pathway. IGF-1 was proved to prohibit TGF-β-induced apoptosis by neutralizing or isolating IGF-BP3 (an IGF-1 binding protein) (Cohen et al., 2000). Numerous experiments suggested that the activation of IGF-1R could block early steps in TGF-β signaling pathway at the level of TGF-β receptors or the activation of Smad (Danielpour and Song, 2006). It was ever revealed that caffeine could promote hair shaft elongation, stimulate hair matrix keratinocyte proliferation, up-regulate IGF-1 gene expression and protein secretion, and down-regulate TGF-β protein secretion. This may also indicate that TGF-β has an inhibitory effect on hair follicle growth, while IGF-1 may stimulate hair follicle cell growth by interacting with it (Fischer et al., 2014).

### 3.3. Wnt/β-catenin signaling pathway

The Wnt/β-catenin pathway plays a central role in hair morphogenesis and cycling during the embryonic stage and adult stage. It is the most important pathway in the proliferation and differentiation of hair follicle stem cells (HFSCs) and promotes the renewal, proliferation and differentiation of HFSCs by binding to the lymphoid enhancer factor (Lef)/T-cell transcription factor in the nucleus (Li et al., 2015). AA, an autoimmune disease in which hair follicles prematurely enter the regressive and telogen stage during the anagen phase, may be related to an abnormal regulatory process of the Wnt/β-catenin pathway (Lee et al., 2021).

### 3.4. Oxidative stress

OS is correlated with the development of many dermal diseases, including AA. OS leads to the loss of immune privilege and facilitates autoimmunity in AA patients by inducing the upregulation of NKG2D ligands (Rajabi et al., 2018). The changes in OS biomarkers such as malondialdehyde, advanced glycation end-products, and ischemic-modified albumin confirm a general pro-oxidative status in AA patients (Peterle et al., 2023). Impaired melanocytes under OS may be the trigger site followed by an attack on hair follicles by the immune system (Xie et al., 2022).

## 4. Gut microbiome and metabolome and AA

### 4.1. Gut microbiome and AA

AA is an autoimmune disease characterized by high levels of proinflammatory cytokines that disrupt the anagen growth phase of hair, eventually leading to spot baldness (Mahmud et al., 2022). According to some relevant studies, the proportion of patients with ulcerative colitis is higher in AA patients than in healthy individuals (Simakou et al., 2019). Although several studies found no or very few significant changes in α or β diversity of the gut microbiota structure of AA patients (Moreno-Arrones et al., 2020; Lu et al., 2021; Rangu et al., 2021; Kang et al., 2022), they had found AA-related microbial biomarkers, including *Megasphaera*, *Achromobacter*, *Lachnospiraceae Incertae Sedis* (Lu et al., 2021), *Ruminococcus bicirculans* (Rangu et al., 2021), *Parabacteroides distasonis* and *Clostridiales vadin BB60* (Moreno-Arrones et al., 2020). Even more, the combination of *Parabacteroides distasonis* and *Clostridiales vadin BB60* could predict the disease status of alopecia universalis with an 80% accuracy (Moreno-Arrones et al., 2020). The above results suggest that there may be a critical link between gut microbiome and the genesis and development of AA, and these relative biomarkers therefore have the potential of being used as early diagnosis and therapeutic targets.

Other evidence regarding the relationship between the intestinal microbiome and AA is as follows: a meta-analysis showed that inflammatory bowel disease is a comorbidity of human AA (Maghfour et al., 2021); genes associated with AA may affect the gut microbiota, inducing the Th1 response that leads to IFN-γ production; C3H/HeJ mice are not only an animal model for AA but also a model for spontaneous colitis (Kopper et al., 2021); and epidemiological data have shown that the incidence of AA is related to diet. The prevalence of AA is 1.7% in the United States, which has a Western diet, and less than 1% in Japan, which has a predominantly soy-based diet, suggesting that dietary nutrients affect the onset and development of AA by altering the intestinal microbiota (Villasante Fricke and Miteva, 2015); comparable results could also be observed in animal models such as mice: the use of vancomycin resulted in the overgrowth of *Lactobacillaceae* in the gut of mice, which consumed and reduced other microflora in the gut. Feeding mice a biotin-deficient diet would cause hair loss, suggesting that *Lactobacillaceae* promote alopecia in a biotin-dependent way (Hayashi et al., 2017). In addition, case reports have shown hair regrowth in two AA patients treated with fecal microbiota transplantation, which supports the hypothesis that the gut microbiome plays a part in the pathophysiology of AA (Xie et al., 2019).

Concerning the relationship between the gut microbiome and AA, a generally accepted interpretation is that accumulating autoreactive T lymphocytes gain tolerance against cell apoptosis, which results in the production of inflammatory cytokines by autoreactive Th1 cells and further leads to prolonged chronic inflammation and hair loss. It is now believed that the functionality of T cells is also influenced by the skin microbiota (Edslev et al., 2020).

T-lymphocyte Tregs are an active part of the peripheral tolerance process and are crucial for the prevention of autoimmune diseases. Tregs are particularly abundant in hair follicles (Raugh et al., 2022). In 2017, a study demonstrated that hair follicle Tregs have the ability to accelerate the proliferation and differentiation of stem cells, which in turn stimulate hair regeneration (Ali et al., 2017). More recent studies have also provided evidence that Treg subpopulations from AA lesions are functionally different from those of healthy tissues (Hamed et al., 2019). Based on these facts, many studies have suggested that short-chain fatty acids (SCFAs) could influence both the number and function of gut Tregs, which have an impact on autoimmune diseases such as AA (Smith et al., 2013; Koh et al., 2016). SCFAs produced by gut bacteria such as *Bacteroides*, *Bifidobacterium* and *Lactobacillus* by fermentation from undigested polysaccharides could enhance the barrier function of the epithelium, lower the permeability of the intestinal barrier and interact with skin receptors to affect or modify dermal commensal bacteria (Martin-Gallausiaux et al., 2021). Low fiber intake and a decrease in SFCA-producing bacteria could cause a decrease in SFCA synthesis by gut bacteria. In this way, gut microbiota may have to do with the pathogenesis of AA.

In addition, dysfunction of the intestinal barrier, including destruction of tight junctions and damage to the mucosal layer, could result in increased intestinal permeability, which has also been termed “leaky gut” syndrome (LGS) (Kinashi and Hase, 2021). Under this circumstance of LGS, gut microbiota may translocate and produce a variety of neurotransmitters that have the ability to pass through the intestinal wall into the bloodstream, causing systemic responses and autoimmune diseases such as AA (De Vos et al., 2022). Nevertheless, the association between increased intestinal permeability and AA still lacks experimental or laboratory evidence, which remains to be further investigated.

### 4.2. Gut metabolome and AA

The gastrointestinal system contains a tremendous number of bacteria, which are capable of producing a huge amount of metabolites. Gut metabolites are molecules with two-sided effects on other organs and the body after entering the circulatory system. That is, the physiological and pathological condition of skin may be related to gut metabolites produced by gut microbiota. Recently, an increasing number of studies on molecular metabolites affecting the host metabolome have emerged, raising questions about the interaction between the gut metabolome and chronic skin diseases.

Human skin and its appendages are target organs of a wide range of neuroendocrine molecules, and we can therefore hypothesize that the abnormal serum levels of some associated neuropeptides and neurohormones have the potential to impact skin health and dermal diseases.

For instance, a study showed that feeding mice a strain of *Lactobacilli* significantly alleviated neurogenic skin inflammation and hair growth inhibition (Arck et al., 2010). This striking result may be associated with several studies that focus on the production of neurotransmitters, including dopamine, GABA and serotonin, by gut microbiota. Resident gut microbiota carries genes encoding digestive enzymes that the host lacks and therefore provide vitamin K, vitamin B12, biotin, folic acid and other micronutrients that may be responsible for hair growth.

As an example, biotin, also known as vitamin B7, is highly dependent on bacterial production. Biotin is a necessary nutrient for human health, especially skin health and hair growth. Decades ago, scientists discovered that biotin deficiency is related to dermatologic diseases such as alopecia. In an experiment, biotin-deficient germ-free (GF) mice developed alopecia, while conventional mice did not. This indicates that in mice fed a biotin-deficient diet, some gut microbiota would deprive the host of biotin and therefore cause alopecia (Hayashi et al., 2017). Experiments on dopamine have also demonstrated the role it plays in the inhibition of hair growth by promoting catagen induction.

Although the association between gut metabolites and AA remains to be further studied, we can still infer that the gut metabolome is an important part of the pathogenesis of AA.

### 4.3. Relationship between the gut microbiome or metabolome and the aforementioned inflammatory pathways

#### 4.3.1. Relationship between the gut microbiome or metabolome and the JAK–STAT signaling pathway

Current studies on the influence of the gut microbiome and metabolome on the JAK–STAT pathway are not sufficient. However, there are experiments showing that parenteral nutrition without going through the gut could change the level of phosphorylated JAK1 and STAT6 (Heneghan et al., 2013). Another study showed that the administration of an elemental enteral diet alone decreased the amounts of IL-4, IL-13, polymeric immunoglobulin receptor (pIgR), and sIgA that were positively associated with JAK1 and STAT6. According to an experiment on the effects of baicalin on atopic dermatitis, the activation of the NF-κB and JAK–STAT pathways in the skin of 2,4-dinitrochlorobenzene (DNCB)-treated mice could be inhibited by baicalin, accompanied by a restoration of the gut microbiome and epidermal barrier function. Furthermore, in these pseudo germ-free (GF) DNCB-treated mice, their dorsal skin thickness and EASI score were reduced, and their serum levels of IgE, histamine, TNF-α and IL-4 were inhibited when they received fecal microbiota from baicalin donors (Wang et al., 2022). LPS, a key outer membrane component of gram-negative bacteria in the gut microbiota, acts as a bridge between inflammation and high-fat diet-induced metabolic syndrome via JAK–STAT and AMPK-dependent cPLA2 expression (Chang et al., 2019).

During the development and use of novel medicines, scientists have revealed their biological effects on the JAK–STAT signaling pathway by affecting the gut microbiota or metabolome. IHS, a kind of spore powder, has been proven to influence the gut microbiota and serum metabolites, further affecting JAK–STAT signaling and the abundance of CD8+ T cells and subsequently showing anti-colorectal cancer (CRC) properties (Yang et al., 2022). MOP, purified from *Moringa oleifera* seeds, can remodel the intestinal mucosal barrier and ameliorate dextran sulfate sodium (DSS)-induced gut microbiota dysbiosis by inhibiting JAK–STAT pathway activation and regulating the gut microbiota and its metabolites (Hong et al., 2022). Moreover, JAK inhibitors such as tofacitinib, which have been put into use for the clinical treatment of hair or scalp diseases such as AA, were observed to influence mucosal immunity and gut microbiota abundance while alleviating the disease (Hablot et al., 2020).

**Figure 1:**
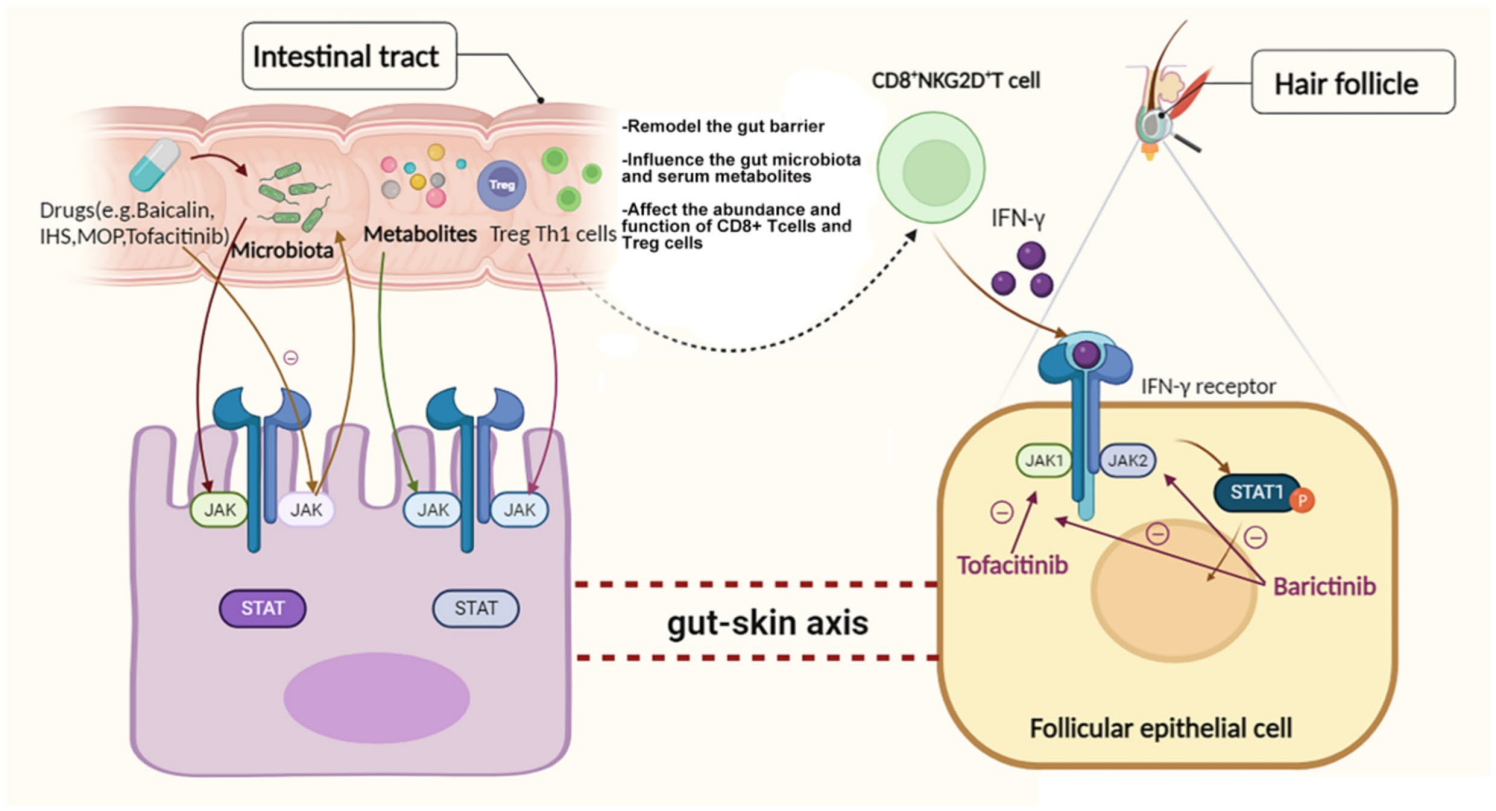
Hypothetical relationships among the gut microbiome or metabolome, the JAK–STAT pathway and AA.

Although evidence for the link between the gut microbiome or metabolome and the change in the JAK–STAT pathway is not strong enough thus far, we can still hypothesize that parenteral nutrition, LPS and some medicines that influence the JAK–STAT pathway could also change the gut microbiota or metabolites to a certain extent, then they further affect the expression level of JAK–STAT pathway in follicular epithelial cell via gut-skin axis (Figure 1).

#### 4.3.2. Relationship between the gut microbiome or metabolome and the TGF-β signaling pathway

Previous experiments have shown that the gut microbiome or metabolome has an impact on the TGF-β pathway. For example, *Bifidobacterium breve* M-16 V may prevent the occurrence of allergies by affecting the gut flora, intestinal epithelial barrier and immune system and has potential beneficial effects on infant health. Even in preterm infants, it can stimulate Treg cells to produce regulatory TGF-β1. In addition, *Bifidobacterium breve* M-16 V could enter the gut and induce the maturation of the epithelial barrier, preventing pathogen infection and translocation and inducing anti-inflammatory processes by promoting the secretion of IFN-γ (Cukrowska et al., 2020). Another study demonstrated that the increased abundance of *Eggerthella* in the fecal samples of patients with premature ovarian insufficiency was reversed when receiving hormone replacement treatment. Furthermore, the abundance of *Eggerthella*, metabolic signatures and serum TGF-β1 levels were positively correlated in the same direction (Jiang et al., 2021). Inhalation of carbon nanotubes (CNTs) simultaneously causes lung inflammation, gut microbiota changes, and TGF-β induction, which indicates a modulatory role of gut dysbiosis and gut-to-lung communication (Bhattacharya et al., 2022). *Lactobacillus plantarum* HNU082 (Lp082) protects the gut mucosal barrier, regulates signaling pathways and increases the expression of MPO, TGF-β1 and TGF-β2 (Wu et al., 2022). Similarly, nisin, cecropin, and *P. chinense* have both the ability to change the abundance and composition of the fish gut microbiota and affect the expression of some anti-inflammatory cytokines, such as TGF-β (Ke et al., 2021). Probiotics such as *Lactobacillus plantarum* AR113 could influence liver generation by increasing the expression of TNF-α, HGF and TGF-β (Xie et al., 2021). All these experimental results indicate that there are correlations between the gut microbiota and the TGF-β signaling pathway; however, functional gain- and loss-of-function experiments are still lacking to confirm the direct relationship between the gut microbiome or metabolome and the TGF-β signaling pathway.

Additionally, several studies had shown that *Lactobacillus* appears to be a key microbes altering the levels of IGF-1 and members of it are well-known producers of SCFAs, which is observed to have direct inhibiting effects on GH production through the cAMP/PKA/CREB pathway (Wang et al., 2013; Jensen et al., 2020). Moreover, a few studies point to a role of microbiota on GH and IGF-1 levels which may be mediated through its production of SCFAs and microbial mimetics or impact on intestinal environment and immune system (Yan and Charles, 2018; Jensen et al., 2020).

#### 4.3.3. Relationship between the gut microbiome or metabolome and the Wnt/β-catenin signaling pathway

Recently, some studies have proven that the gut microbiome plays a role in the onset of several diseases, such as colorectal cancer, by affecting the associated Wnt/β-catenin signaling pathway. Many studies have cast light on a gut metabolite called short-chain fatty acid (SCFA) butyrate, which serves as a histone deacetylase (HDAC) inhibitor. A number of studies have indicated that butyrate has the ability to induce Wnt/β-catenin activity and apoptosis. It is now confirmed that hyperactivation of the Wnt/β-catenin pathway is a major contributor to colorectal carcinogenesis (Tian et al., 2018). *Portulaca oleracea* extract (POE) treatment inhibits the development of colorectal carcinoma in mice and causes changes in the gut microbiota, among which 20 differential microbiota may participate in CRC development via the Wnt pathway (Yi et al., 2022). *Lactobacillus* species also inhibit the progression of CRC by modulating the Wnt/β-catenin pathway (Ghanavati et al., 2020). For intestinal stem cells (ISCs), gut microbiota and metabolites trigger a series of chain reactions involving 5-HT and PGE2, eventually promoting the regeneration of ISCs (Zhu et al., 2022). The Chinese herbal medicine Qingchang Wenzhong decoction has been proven to modulate gut microbiota and may further activate Wnt/β-catenin signals, thus promoting ISC renewal (Sun et al., 2021). Another experiment indicated that gut microbiota dysbiosis and hypoxia can inhibit low-density lipoprotein receptor-related protein 6 (LRP6) and the Wnt/β-catenin pathway, while drugs targeting LRP6 can protect the intestinal barrier and restore the gut microbiota by regulating the Wnt/β-catenin pathway (Liu et al., 2022). Since AA showed an abnormality in the regulation process of the Wnt/β-catenin pathway and a link with the intestinal system, we believe that the gut microbiota and metabolome are also associated with this abnormality.

### 4.4. Relationship between the gut microbiome or metabolome and OS

Recent experiments have revealed that in the presence of microbiota, gut epithelial cells generate physiological levels of OS and in turn interfere with the composition and function of the microbiota. Since the homeostasis of the gut barrier is affected by the redox ability of the gut microbiota, the permeability of the gut would increase, probably leading to LGS and causing autoimmune diseases such as AA. Emerging evidence has displayed the close interaction between OS and the gut microbiome or metabolome, which is involved in the pathogenesis of neurodegeneration and neuroprotection (Shandilya et al., 2022), inflammatory skin diseases (Ni et al., 2022), and inflammatory bowel disease (Bourgonje et al., 2020). According to recent opinions, gut dysbiosis could influence the redox state and cause inflammation, ultimately leading to inflammatory dermal diseases or previous systemic diseases via the gut-skin or gut-brain axis-related response (Ni et al., 2022). It was revealed by an MRL/lpr mouse model for autoimmune diseases that gut microbiome dysbiosis is associated with increased colonic OS and barrier dysfunction (Wang et al., 2021). Supplementation with a healthy microbiome and specifically *Lactobacillus reuteri* could reverse the redox balance and inhibit the proliferation of CRC cells via the induction of selective protein oxidation (Bell et al., 2022).

### 4.5. Relationship between the microbiota of hair follicles and the modulation of inflammatory process

There is a significant presence of microbial colonization within the hair follicle funnel, accompanied by an ongoing microbiota-immune system crosstalk at the scalp barrier. Furthermore, the microbiota extends beneath the hair follicle funnel, particularly abundant in *Propionibacterium acnes* and *Staphylococcus epidermidis,* which may be responsible for conferring immune privilege (Skogberg et al., 2017). A study analyzing the microbiota of the hair follicle of patients with AA revealed that the diversity of hair follicle microbiota in AA patients is higher, with an increase in *Propionibacterium acnes*, and a decrease in *Staphylococcus epidermidis* (Lousada et al., 2021). They further uncovered that *Prevotella copri*, which is confirmed to be involved in the pathogenesis of rheumatoid arthritis (Alpizar-Rodriguez et al., 2019), expressed most abundantly in the subcutaneous tissue of AA, suggesting that *Prevotella copri* evokes the autoimmune disturbance of hair follicles in AA. Additionally, external stimuli can impact the activation state of the immune system dysregulation of the dynamic balance that maintains the immune privilege state for hair follicles. This dysregulation activates abnormal signaling pathways, modulating inflammatory process, resulting in damage to the microecology of stem cells and hindering hair regeneration (Constantinou et al., 2021). More interestingly and unexpectedly, skin-resident bacteria increases the glutamine metabolism in keratinocytes by induction of hypoxia through HIF-1α signaling, further to enhance the regeneration of skin and hair follicles (Wang et al., 2023).

## 5. Conclusion and perspectives

**Figure 2:**
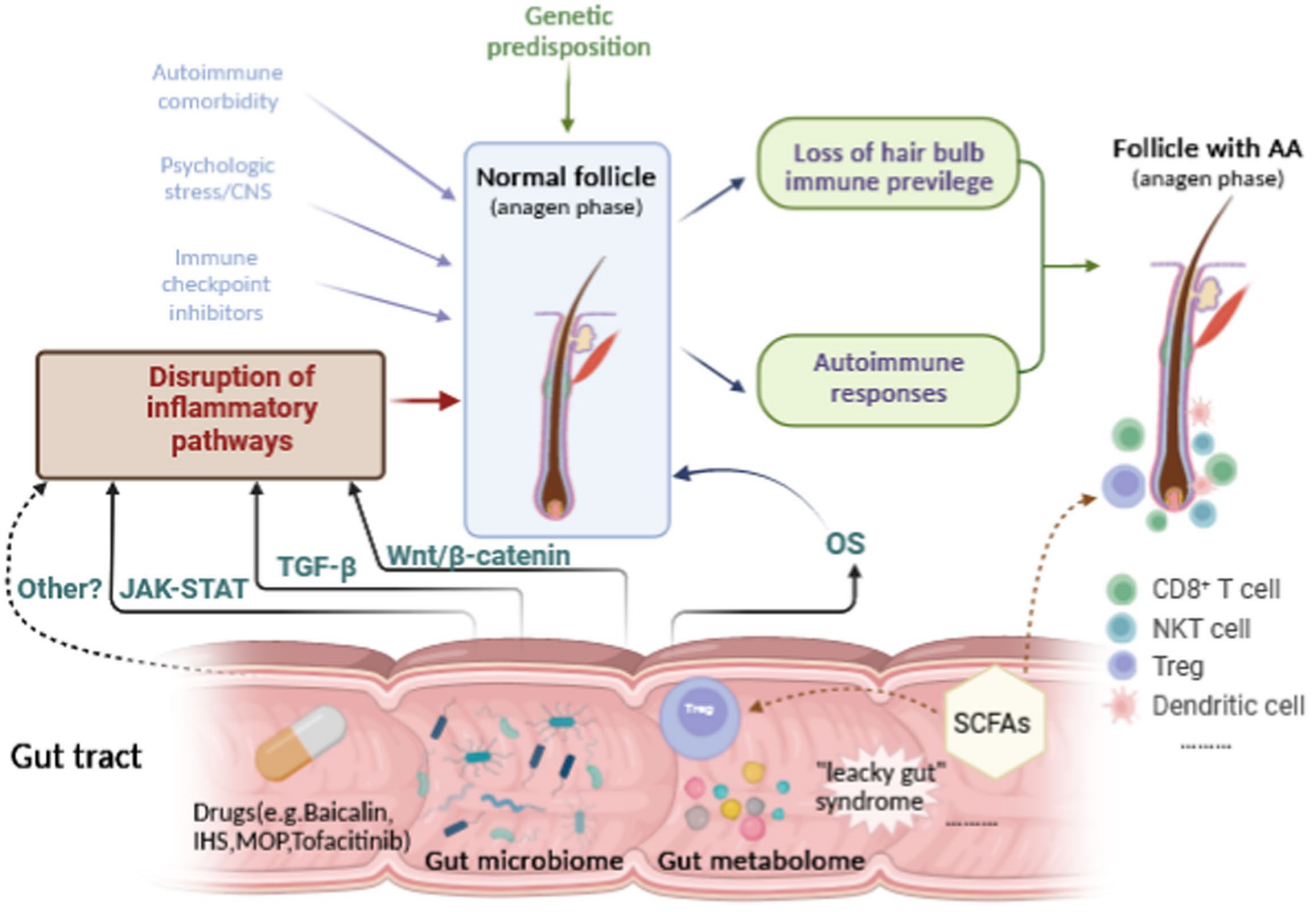
Hypothetical relationships among the gut microbiome or metabolome, the inflammatory pathways, OS and AA.

Alopecia areata is an autoimmune dermatological disease with complex and unclear etiology that causes great physical and psychological damage to patients. With more attention being paid to the gut-skin axis in recent years, we now believe that the gut microbiome and metabolome are likely to play a part in the development of AA. Evidence has increasingly demonstrated that the gut microbiome and metabolome are involved in the pathogenesis of AA. Although there are no direct and conclusive studies to confirm the specific mechanisms between the gut microbiome or metabolome and OS and inflammatory signaling pathways in AA, accumulating studies in other diseases have validated the effect of the gut microbiome or metabolome on the JAK–STAT, TGF-β, Wnt/β-catenin or other signaling pathways, which are also critical pathways involved in the etiopathogenesis of AA (Figure 2). However, many complementary studies need to be conducted to elucidate the complex interactions between the gut microbiota or metabolites and AA.

With the more and more in-depth researches on the impact of gut microbiota or metabolites, the application of interventional approaches, such as probiotics, prebiotics, fermenting microbes and FMT therapies as routine or adjuvant therapeutic strategies, hold promise for novel and effective treatments. Although there were only case reports of FMT from healthy donors by restoring the homeostasis of the gut flora in AA patients (Xie et al., 2019), accumulating clinical evidences support its potential efficacy in treatments of tumors (Davar et al., 2021; Routy et al., 2023), autoimmune diseases (Huang C. et al., 2022), inflammatory bowel diseases (Sokol et al., 2020), especially the latter two that their pathogenesis is also related to JAK–STAT signaling pathway. For applications of probiotics or prebiotics in the field of hair loss, it was reported that cheonggukjang probiotic product, fermented by *Bacillus*, *Lactobacillus*, *Leuconostoc* and *Enterococcus faecium*, could promote hair growth and reverse hair loss (Park et al., 2020). Furthermore, use of probiotics, such as heat-killed *Lacticaseibacillus paracasei* GMNL-653 (Tsai et al., 2023), fermenting microbes (Mayer et al., 2023) in hair care cosmetics, also exhibited the activities of balancing hair microbiota and promoting hair growth. To our excitement, dietary fiber and probiotics improve host response to immune checkpoint blockade in tumor growth (Si et al., 2022; Bender et al., 2023)in addition to their robust efficacy in remodeling intestinal health (Sanders et al., 2019). Therefore, we are fairly confident that probiotics, prebiotics and FMT therapies will be a potential adjuvant therapeutic alternative for AA.

Still, microbial interventions also raise some questions and challenges. First all, although some AA-related microbial biomarkers were reported, there still lacks precise gut microbial biomarkers in AA, especially in alopecia universalis and alopecia totalis in a large number scale of specimens. Furthermore, the functions of these reported biomarkers were not validated by gain- or loss-functional experiments. In addition to *Lactobacillus* and *Bifidobacterium* species, and prebiotics of fructooligosaccharides and galactooligosaccharides, whether there are specific probiotics or prebiotics for AA, it also remains unknown. Secondly, expert consensus statements on FMT (Ng et al., 2020; National Institute of Hospital Administration, et al., 2022), probiotics (Hill et al., 2014) and (Gibson et al., 2017) have been published to guide the clinical use of microbial interventions. However, the administration time, the optimal dose and duration of these treatments for AA have not been completely determined. Therefore, it is essential to actively explore the mechanisms underlying the microbiota-gut-skin cross talk in AA, to precisely pinpoint the functions of the microbial products and their possible host interactions, and to strictly carry out the randomized controlled trials for these microbial interventions in AA.

## Author contributions

ZL: Data curation, Visualization, Writing – original draft. XL: Conceptualization, Supervision, Writing – review & editing.
